# Catheter Ablation of Idiopathic Premature Ventricular Contractions and Ventricular Tachycardias Originating from the Vicinity of Endocardial and Epicardial Mitral Annulus

**DOI:** 10.1371/journal.pone.0080777

**Published:** 2013-11-27

**Authors:** Li Yue-Chun, Zheng Cheng, Hong Jun, Cheng Jun-Hua, Zhao Jing-Lin, Lin Jia-Feng

**Affiliations:** Department of Cardiology, Second Affiliated Hospital of Wenzhou Medical College, Wenzhou, China; Scuola Superiore Sant'Anna, Italy

## Abstract

**Background:**

Several previous reports have revealed that idiopathic ventricular arrhythmias (VAs), including premature ventricular contractions (PVCs) and ventricular tachycardias (IVTs), can originate from endocardial mitral annulus (ENDO MA). However, these data are limited to ENDO MA VAs, and little is known about the electrocardiographic (ECG) characteristics and the efficacy of radiofrequency catheter ablation (RFCA) via the coronary venous system for the VAs arising from the epicardial MA (EPI MA).

**Methodology/Principal Findings:**

Characteristics of body surface electrocardiogram and electrophysiologic recordings were analyzed in 21 patients with symptomatic PVCs/IVTs originating from the vicinity of MA. Among 597 patients with PVCs/IVTs, the incidence of VAs originating from the ENDO and EPI MA was 3.52% (21 cases). Eleven (52%) from the ENDO MA, and 10 (48%) from the EPI MA. There were different characteristics of ECG of PVCs/VT originating from the ENDO and EPI MA. The prolonged pseudodelta wave time and intrinsicoid deflection time in lead V2 and the precordial maximum deflection index reliably differentiated EPI MA VAs from ENDO MA VAs with high sensitivity and specificity. Successful RFCA in 18 patients could be achieved (85.7% acute procedural success).

**Conclusions/Significance:**

ECG characteristics of PVCs/VTs originating from the different portions of the MA are different, and can help regionalize the origin of these arrhythmias. RFCA within the coronary venous system was relatively effective and safe for the PVCs/IVTs and should be seen as an alternative approach, when the MA PVCs/IVTs could not be eliminated by RFCA from the endocardium.

## Introduction

The majority of idiopathic left ventricular arrhythmias (VAs), including idiopathic premature ventricular contractions (PVCs) and ventricular tachycardias (IVTs), have an aortic root or left ventricular (LV) septum origin, but some originate from the endocardium of the left ventricular outflow tract (LVOT), mitral annulus (MA), aorto-mitral continuity and LV epicardium [1]–[8]. Endocardial radiofrequency catheter ablation (RFCA) is the common approach for ablation of the VAs [1]–[7]. However, some that arise from the epicardial or subendocardial portion are remote from the LV endocardium and can not be ablated from the ventricular endocardium [8], [9]. When the site of origin is near the left coronary veins, RF energy can be delivered directly in veins [8]–[11]. Several previous reports have revealed that the VAs can originate from the endocardial mitral annulus (ENDO MA), as confirmed by pace mapping and successful RFCA from the endocardium [5], [6]. However, these data are limited to endocardial mitral annular VAs, and little is known about the electrocardiographic (ECG) characteristics and the efficacy of RFCA via the coronary venous system for the VAs arising from the epicardial mitral annulus (EPI MA). The aim of this study was to investigate whether ECG characteristics can be used to identify the arrhythmia origin and guide catheter ablation from the ENDO and EPI MA.

## Methods

### Study population

From July 2006 to March 2012, a total of 597 patients (252 men and 345 women; age 46.88±17.18 years [mean ± SD]) without structural heart disease were presented for catheter ablation for PVCs/IVTs in our hospital. Twenty-one of the 597 patients were found to have idiopathic PVCs/IVTs originating from the vicinity of the ENDO and EPI MA and are the focus of the present study. All patients were verified as having no structural heart disease, including coronary artery disease, valvular heart disease, congenital heart disease, left ventricle hypertrophy, and right ventricle abnormalities by routine biochemistry tests, X-ray, color echocardiography examination, exercise electrocardiogram testing, and/or cardiac catheterization with coronary angiography. Before RFCA, a 12-lead ECG was obtained, and 24 h of ambulatory ECG monitoring (Holter) was carried out at least once. The ECG was monitored for 24 h just before catheter ablation. A pace mapping study was also performed in 12 control subjects (7 men and 5 women; age 38.71±11.65 years) without structural heart disease after successful ablation of their original atrioventricular reentrant tachycardia to determine the ECG characteristics of idiopathic PVCs/IVTs originating from the ENDO and EPI MA.

### Ethics Approval

Ethical approval was obtained from the Ethics Committee of the Second Affiliated Hospital of Wenzhou Medical College, and All participants consented to the experimental procedures. Written informed consent was obtained from each participant.

### Inclusion criteria

The selection criteria of patients were the following reasons: (1) frequent or consecutive PVC occurrence, the average PVC count ≥10000 times/24 h; (2) inability of the patient to tolerate PVCs/IVTs or unsuccessful treatment with at least one antiarrhythmic drugs; and (3) consent for the catheter ablation procedure.

### Electrophysiologic study and RFCA

Anti-arrhythmic drugs were withdrawn in all patients at least five half-lives before ablation, with the exception of amiodarone that was withdrawn eight weeks before intervention. Standard multielectrode catheters were inserted through femoral veins under fluoroscopic guidance. Programmed electrical stimulation was performed from right ventricular apex at basic drive cycle lengths of 600, 500, and 430 msec, delivering a maximum of three extrastimuli. If the clinical arrhythmia did not occur spontaneously and was not induced in the baseline, intravenous isoproterenol (2–4 µg/min) was administered to induce arrhythmia. A 12-lead surface ECG was monitored and recorded on a multichannel oscilloscopic recorder. Detailed activation mapping and pacing mapping were performed using a 4-mm-tip ablation catheter introduced from the right femoral vein and right femoral artery. The activation time was measured from the onset of the bipolar electrogram (earliest positive or negative deﬂection) of the distal bipole of the mapping catheter to the earliest onset of the QRS complex in any of the 12 ECG leads. Activation times and pace-map match were measured/judged visually by 2 independent observers, with each of the 12 ECG leads assessed for the QRS vector and major notching or deﬂections from baseline for the pace-map assessment. If activation mapping and pace mapping indicated that the right ventricle was not the likely source of PVC/IVTs, mapping of the aortic sinus of Valsalva (ASOV), LVOT and ENDO MA was performed via the retrograde aortic route. If the pace mapping and activation mapping did not indicate a focus inside the ASOV or LVOT or ENDO MA, and the left-sided PVC/IVTs can not be abolished by RF application from the ASOV or LVOT or ENDO MA, mapping and ablation was performed within the coronary venous system. Coronary venous anatomy was defined by coronary venous angiography prior to mapping.

The target site for RFCA was determined by activation mapping (earliest local activation time preceding the earliest surface QRS by ≥20 msec) in patients with frequent PVCs/sustained IVT, and by pace mapping (≥11/12–lead concordance of major and minor deflections between the pace map and the clinical PVCs) in those with infrequent arrhythmia. Coronary angiography was performed prior to and after RF application within the coronary venous system. After the target site was located, RFCA was applied in all patients by using irrigated-tip catheter (Endocardial ablation: 43°C, 30 W, 20 ml/min; Epicardial ablation: 43°C, 25 W, 30 ml/min) with the three-dimensional mapping (Ensite NavX system or Carto XP or Carto 3 system) or a conventional catheter under temperature control (Endocardial ablation: target temperature 60°C, maximum power 50 W; Epicardial ablation: target temperature 60°C, maximum power 20 W). If there is too high impedance within coronary venous system to deliver RF energy using conventional catheter, we will choose to use an irrigated-tip catheter. If the PVCs/IVTs were terminated within 10 s or more PVCs and/or nonsustained VT occurred during ablation at the target site, additional current was applied for another 60 to 180 s. Acute procedural successful ablation was defined as complete elimination of spontaneous or inducible VAs. Programmed electrical stimulation was repeated at 30 min after the last application of RF energy to confirm the absence of inducible VAs before removing all catheters and sheaths. If PVCs/IVTs did not terminate within 10 s, the RF energy application was terminated and another target site was sought.

### Definition of PVCs/IVTs originating from the ENDO and EPI MA

**Figure 1:**
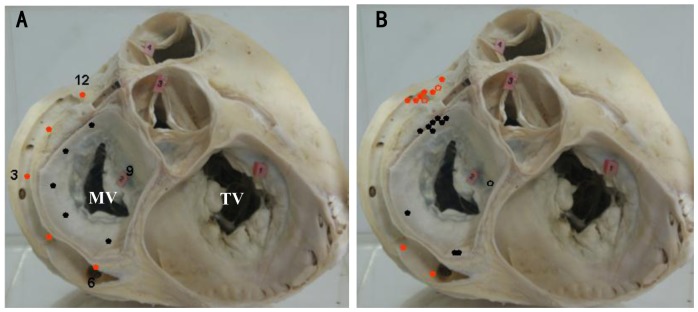
Posterior anterior view of the endocardial and epicardial mitral annulus. A: The different pacing sites represented by the red dots (epicardial mitral annulus sites) and black dots (endocardial mitral annulus sites) in pace mapping study; B: The distribution of the origin of idiopathic ventricular arrhythmias, represented by red dots (epicardial mitral annulus origin) and black dots (endocardial mitral annulus origin). The successful ablation sites were indicated by the red and black solid dots, and the unsuccessful ablation sites were indicated by the red and black hollow dots.

PVCs/IVTs were considered to originate from the vicinity of ENDO MA, based on (1) the characteristic of MA location and motion (when viewed in the right and left anterior oblique fluoroscopic views after successful RFCA); (2) the local endocardial recordings. The ratio of the atrial to ventricular electrograms at the ablation site was <1, and the amplitudes of the atrial and ventricular electrograms were≧0.08 and >0.5 mV at the ablation site, respectively. PVCs/IVTs were considered to originate from the vicinity of EPI MA, based on the venogram images and the local electrograms recorded at the left coronary veins ablation site. In the left anterior oblique (LAO) projection, the MA was viewed as a clock face, that was divided into 6 portions (Figure 1): anteroseptum (approximately 10 o'clock to 12 o'clock), midseptum (approximately 8 o'clock to 10 o'clock), posteroseptum (approximately 6 o'clock to 8 o'clock position), anterolateral portion (approximately 12 o'clock to 2 o'clock position), lateral portion (approximately 2 o'clock to 4 o'clock position), posterolateral portion (approximately 4 o'clock to 6 o'clock position).

### ECG measurements

The following ECG features were assessed in patients with PVCs/IVTs originating from the ENDO and EPI MA: (1) The QRS morphology of the PVCs/IVTs in all 12 leads, (2) the site of R-wave transition in the precordial leads, (3) the axis deviation, (4) the “notching” of the QRS complex in the inferior leads, (5) the QRS complex duration, (6) the pseudo delta wave (PdW, the interval from the beginning of the QRS complex to the earliest fast deflection in any precordial lead) time, (7) the intrinsicoid deflection time (IDT, the interval from the beginning of the QRS complex to the peak of the R wave in V2), and the maximum deflection index (MDI, IDT divided by the QRS duration) were assessed as previously described [12]. ECGs were reviewed by 2 investigators blinded to the site of origin; discrepancies were adjudicated by a third investigator. Capital letters (Q,R,S) refer to relatively high-amplitude waves (>5 mm). Conversely, lowercase letters (q,r,s) refer to relatively low-amplitude waves(≤5 mm).

### Pace mapping study

The pace mapping study was performed in the 12 control subjects. A 7-Fquadripolar catheter was used. A single electrical stimulus was delivered during the end diastole in a bipolar fashion at an output just greater than the diastolic threshold from the distal electrode pair (with the distal electrode as the cathode). The catheter sites were confirmed by multi-plane ﬂuoroscopy, and pacing was performed at the ENDO and EPI MA in each patient (Figure 1A). Coronary venous anatomy was defined by coronary venous angiography prior to pace mapping. The pacing protocol was performed after a written informed consent was obtained.

### Postablation follow-Up

After RFCA, all patients underwent a 48-hour ECG monitoring. Holter was carried out 1 week after RFCA. Patients were not given any antiarrhythmic drugs after RFCA, and underwent color echocardiography and Holter examination 3 month after RFCA. ECG and 24-hour ECG monitoring were performed whenever the patient had symptoms suggestive of recurrence of VAs.

### Statistical Analysis

All values were expressed as mean value ± standard deviation (SD). The continuous variables were compared with a Student's t-test for two groups and with analysis of variance (ANOVA) for >2 groups. The categorical data were compared with a Fisher's exact test. The sensitivity, specificity, and positive and negative predictive accuracies of the ECG characteristics to localize the site of origin of PVCs/IVTs were calculated. All tests were 2-tailed, and a value of P <0.05 was considered statistically significant.

## Results

### Study population

Among patients with PVCs/IVTs, the incidence of VAs originating from the vicinity of ENDO and EPI MA was 3.52% (total 21 patients, 13 men and 8 women; mean age 50.09±13.58 years). The clinical characteristics of the 21 patients included in the study are shown in Table 1. The past medical history was significant for hypertension in 5 patients, diabetes in 3 patients. Symptoms consisted of palpitations (100%), presyncope (9.5%), syncope (4.8%), and chest pain (19.0%). The median duration of symptoms prior to the ablation procedure was 19.5 months (range 3–48). All patients in this study had failed prior antiarrhythmic therapy with 1.52±0.51 drugs, including amiodarone therapy in 4 patients (19.0%). No patients had a family history of sudden cardiac death. The mean left ventricular ejection fraction (LVEF) was 60.0±7.1% (2 patients with a markedly decreased LVEF), and the left ventricular end-diastolic internal diameter (LVEDd) was 49.7±6.5 mm (4 patients with a markedly dilated left ventricle).

**Table 1:** Baseline patient characteristics.

Patient	Age (y)	Sex	PVC count (number/24 h)	Symptoms	Symptom duration(M)	AADs used	Comorbidities	LVEF (%)	LVEDd (mm)
1	49	M	10971	palpitation, chest pain	10	Beta-blocker	none	65	48
2	32	M	13054	palpitation	36	Beta-blocker, Mexiletine	none	72	45
3	55	F	27146	palpitation	18	Beta-blocker, Mexiletine	none	46	65
4	50	F	18709	palpitation	30	Beta-blocker	diabetes	52	59
5	37	M	39672	palpitation	7	Beta-blocker, Propafenone	none	67	52
6	59	F	21980	palpitation	12	Beta-blocker	diabetes	65	53
7	33	M	18280	palpitation,presyncope	24	Beta-blocker, Propafenone	none	61	46
8	58	M	15731	Palpitation	6	Beta-blocker	none	63	45
9	57	M	18058	Palpitation	48	Beta-blocker, Amiodarone	hypertension	41	52
10	46	M	12090	Palpitation	6	Beta-blocker	none	60	43
11	66	F	11925	Palpitation, presyncope	10	Beta-blocker	hypertension	59	48
12	64	M	17235	Palpitation	15	Beta-blocker	none	62	46
13	65	M	18563	Palpitation	43	Beta-blocker, Amiodarone	none	59	46
14	42	F	16802	Palpitation, chest pain	6	Beta-blocker	diabetes, hypertension	61	46
15	21	M	26944	palpitation	24	Beta-blocker, Propafenone	none	57	62
16	80	M	17906	palpitation, chest pain	12	Beta-blocker, Amiodarone	hypertension	61	47
17	39	M	15972	palpitation	32	Beta-blocker, Propafenone	none	65	45
18	43	F	14578	palpitation	36	Beta-blocker, Mexiletine	none	66	43
19	54	M	11080	palpitation	9	Beta-blocker	none	64	45
20	50	F	31925	palpitation, syncope	24	Beta-blocker, Amiodarone	hypertension	53	60
21	52	F	13740	palpitation, chest pain	3	Beta-blocker	none	62	48
Mean ± SD	50.09 ±13.58		18683±7310		19.5±13.4			60.0±7.1	49.7±6.5

Y = years, M = Months, AADs = antiarrhythmic drugs, LVEF = left ventricular ejection fraction, LVEDd = left ventricular end-diastolic internal diameter.

### Baseline 24-Hour ECG Monitoring

The mean PVC burden during the preoperative 24 h of ambulatory Holter monitoring was 18683±7310 (range 10971–39672): 15 patients (71.4%) had single isolated PVC, 2 patients (9.5%) had ventricular couplets, 1 patient (4.8%) had non sustained monomorphic VT (defined as ≧ 3 consecutive PVCs, duration ≦ 30 s), 3 patients (14.3%) had sustained monomorphic VT (duration ≧ 30 s) (Table 1). The PVCs were monomorphic in all patients.

### Electrophysiologic findings and effect of RFCA

PVCs/IVTs arise from the ENDO and EPI MA in 21 patients, including 11 (52%) from the ENDO MA (7 from anterolateral portion, 1 from posterolateral portion, 1 from midseptum, 2 from posteroseptum), and 10 (48%) from the EPI MA (8 from anterolateral portion, 1 from posterolateral portion, 1 from posteroseptum). No PVCs/IVTs originated from the anteroseptal and lateral portion of MA. Figure 1B and Table 2 list the detailed VA origin from the ENDO and EPI MA. The PVCs/IVTs occurred spontaneously in 15 patients and were induced by bolus injection of isoproterenol (2 µg) in 6 patients during the electrophysiologic study. The local ventricular activation time recorded at successful ablation target sites that preceded the onset of the QRS complex was 33.9±5.0 ms (Table 2). Successful RFCA in 18 patients could be achieved (85.7% acute procedural success). Endocardial ablation was successful in 10 of 11 patients. Ablation could not be applied in the midseptum of the ENDO MA in the remaining 1 patient because the best pace and activation mapping site was close to the His bundle. Epicardial ablation was successful in 8 of 10 patients. In the remaining 2 patients, RF applications in the distal great cardiac vein were limited by high impedance. No complications occurred during the mapping or ablation procedure. There was no significant difference in the local ventricular activation time relative to the QRS onset (−34.7±6.0 ms VS −33.3±4.2 ms), number of RF lesions (3.7±1.8 VS 3.0±1.5), and acute procedural success rate (80% VS 90.91%) between the EPI MA and the ENDO MA origins (Table 3). The RF application duration was significantly shorter and the procedure duration and the fluoroscopy time were significantly longer for the VAs with EPI MA origin than for those with ENDO MA origin (p<0.01) (Table 3).

**Table 2:** The RFCA outcome.

Patient	Origin	Mapping technique	EAT (ms)	Pace mapping (12 lead concordance)	Ablation outcome	RF lesions prior to success	Procedural complication	Recurrent	Follow up time (M)
1	AL-EPI MA	EAT+Pace	36	11	success	3	no	no	51
2	AL-EPI MA	EAT+Pace	31	12	success	2	no	no	20
3	AL-EPI MA	EAT+Pace	37	12	failure	7	no	-	20
4	AL-EPI MA	EAT+Pace	47	12	success	1	no	no	3
5	AL-EPI MA	EAT+Pace	33	12	success	3	no	no	30
6	AL-EPI MA	EAT+Pace	37	12	success	3	no	yes	24
7	AL-EPI MA	EAT+Pace	26	11	failure	6	no	–	6
8	AL-EPI MA	EAT+Pace	36	12	success	3	no	no	9
9	PL-EPI MA	EAT+Pace	37	12	success	5	no	no	8
10	PS-EPI MA	EAT+Pace	27	10	success	4	no	no	15
11	PS-ENDO MA	EAT+Pace	31	12	success	3	no	no	18
12	PS-ENDO MA	EAT+Pace	32	10	success	7	no	no	24
13	AL-ENDO MA	EAT+Pace	31	11	success	4	no	no	32
14	AL-ENDO MA	EAT+Pace	35	12	success	3	no	no	9
15	PL-ENDO MA	EAT+Pace	33	12	success	3	no	no	18
16	AL-ENDO MA	EAT+Pace	30	11	success	2	no	no	9
17	AL-ENDO MA	EAT+Pace	45	12	success	2	no	no	42
18	AL-ENDO MA	EAT+Pace	32	11	success	3	no	no	18
19	MS-ENDO MA	EAT+Pace	30	12	failure	1	no	–	20
20	AL-ENDO MA	EAT+Pace	34	12	success	2	no	no	4
21	AL-ENDO MA	EAT+Pace	33	12	success	3	no	no	4
Total			33.9±5.0	11.6±0.7	Procedural success rate: 85.7%	3.28±1.76	Procedural complication rate: 0	Recurrent rate (%): 5.5	18.2±12.6

RFCA: radiofrequency catheter ablation; EAT: earliest activation time; AL-EPI MA: anterolateral portion of the epicardial mitral annulus; PL-EPI MA: posterolateral portion of the epicardial mitral annulus; PS-EPI MA: posteroseptal portion of the epicardial mitral annulus; PS-ENDO MA: posteroseptal portion of the endocardial mitral annulus; AL-ENDO MA: anterolateral portion of the endocardial mitral annulus; PL-ENDO MA: posterolateral portion of the endocardial mitral annulus; MS-ENDO MA: midseptal portion of the endocardial mitral annulus.

**Table 3:** Procedural Details.

Origin	V-QRS (ms)	No.of RF Lesions	RF Duration (s)	Procedure time (min)	Fluoroscopy time (min)	Acutely successful ablation procedure (%)
EPI MA (n = 10)	−34.7±6.0	3.7±1.8	135.9±33.9	82.9±23.8	16.4±4.9	8 (80.00)
AL-EPI MA (n = 8)	−35.4±6.0	3.5±2.0	131.5±38.8	77.1±18.5	13.3±4.1	6 (75.00)
ENDO MA (n = 11)	−33.3±4.2	3.0±1.5	368.8±74.4*	58.6±13.2*	9.6±4.1*	10 (90.91)
AL-ENDO MA (n = 7)	−34.3±5.0	2.7±0.8	306.3±56.4^▴^	53.0±10.3^▴▴^	8.7±3.6^▴^	7 (100.00)

RF: radiofrequency; V-QRS: local ventricular activation time relative to the QRS onset; EPI MA: epicardial mitral annulus; AL-EPI MA: anterolateral portion of the epicardial mitral annulus; ENDO MA: endocardial mitral annulus; AL-ENDO MA: anterolateral portion of the endocardial mitral annulus. *P<0.01 versus EPI MA; ^▴^P<0.01 versus AL-EPI MA; ^▴▴^ P<0.05 versus AL-EPI MA.

### Comparison between three-dimensional mapping and conventional mapping in the ablation of PVCs/IVTs originating from the mitral annulus

Fifteen of 21 patients underwent the electrophysiologic study using three-dimensional mapping system (Ensite NavX system or Carto XP system or Carto 3 system), and the remaining 6 patients using conventional fluoroscopy guided mapping techniques. There was no significant difference in the local ventricular activation time relative to the QRS onset, number of ablation lesions, acute success, follow up duration, and freedom of VAs at follow up between the patients using three-dimensional mapping and conventional mapping (Table 4). No complications occurred during the three-dimensional and conventional mapping.

**Table 4:** Comparison between three-dimensional mapping and conventional mapping in the ablation of PVCs/IVTs originating from the endocardial and epicardial mitral annulus.

	Three-dimensional activation mapping (n = 15)	Conventional activation mapping (n = 6)	P-Vaule
V-QRS (ms)	34.7±5.3	32.2±4.1	0.32
RF Lesions (no.)	3.1±1.2	4.0±2.5	0.26
Acute success (no.)	14(93.3%)	4(66.7%)	0.18
Procedural complication (no.)	0	0	–
Follow up duration (months)	18.1±14.7	18.17±5.8	0.93
Freedom of VAs at follow up (no.)	14(100%)	3(50%)	0.22

V-QRS: local ventricular activation time relative to the QRS onset; RF: radiofrequency; VAs: ventricular arrhythmias.

### ECG characteristics

#### General characteristics

**Figure 2:**
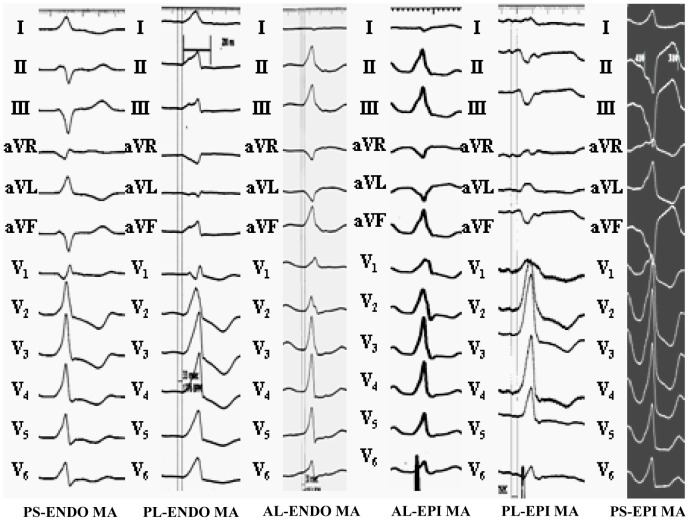
Representative 12-lead electrocardiograms of premature ventricular contractions originating from the endocardial and epicardial mitral annulus (MA). PS-ENDO MA: posteroseptal portion of the endocardial MA; PL-ENDO MA: posterolateral portion of the endocardial MA; AL-ENDO MA: anterolateral portion of the endocardial MA; AL-EPI MA: anterolateral portion of the epicardial MA; PL-EPI MA: posterolateral portion of the epicardial MA; PS-EPI MA: posteroseptal portion of the epicardial MA.

**Figure 3:**
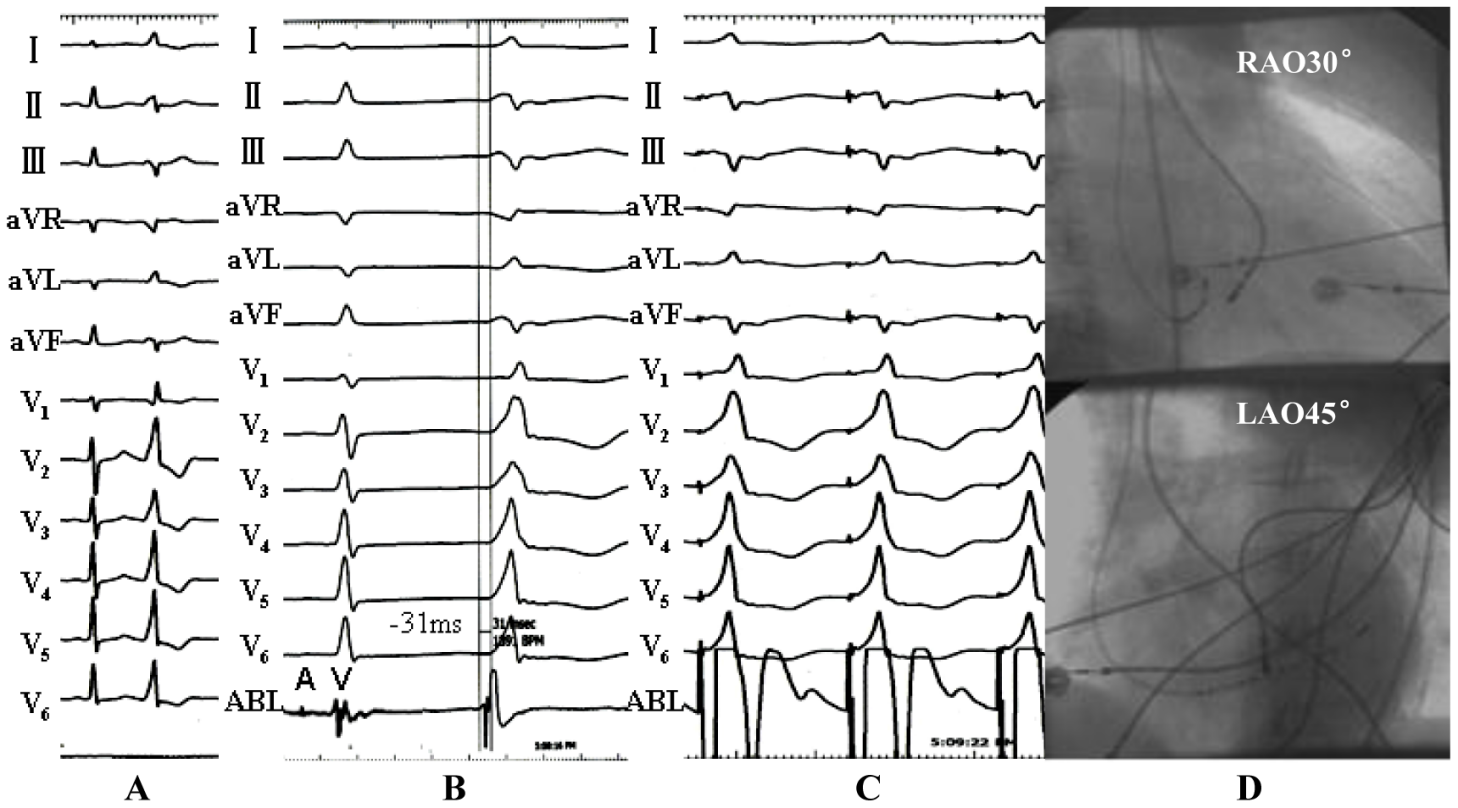
Example of a successful ablation of idiopathic premature ventricular contraction originating from the posteroseptal portion of the endocardial mitral annulus. (A) The surface ECG characteristic. (B) The local ventricular activation time recorded at the successful ablation site that preceded the onset of the QRS complex was 31 ms. (C) Pace map at the ablation site. (D) The ﬂuoroscopic position of the ablation catheter site.

**Figure 4:**
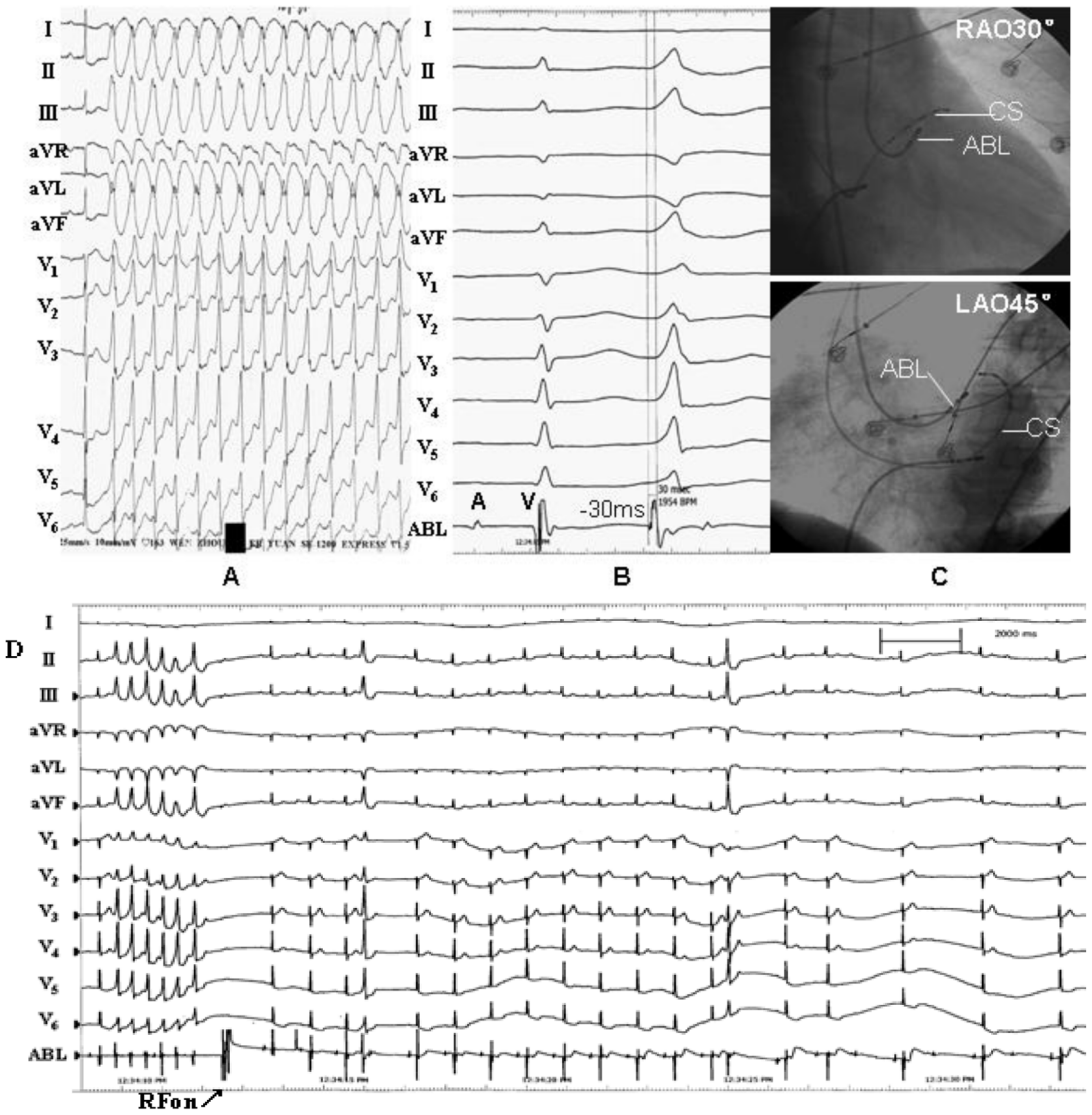
Example of a successful ablation of idiopathic ventricular tachycardias originating from the anterior portion of the endocardial mitral annulus. (A) The surface ECG characteristic. (B) The local ventricular activation time recorded at the successful ablation site that preceded the onset of the QRS complex was 30 ms. (C) The ﬂuoroscopic position of the ablation catheter site. (D) Termination of ventricular tachycardias during RF application at the site.

**Figure 5:**
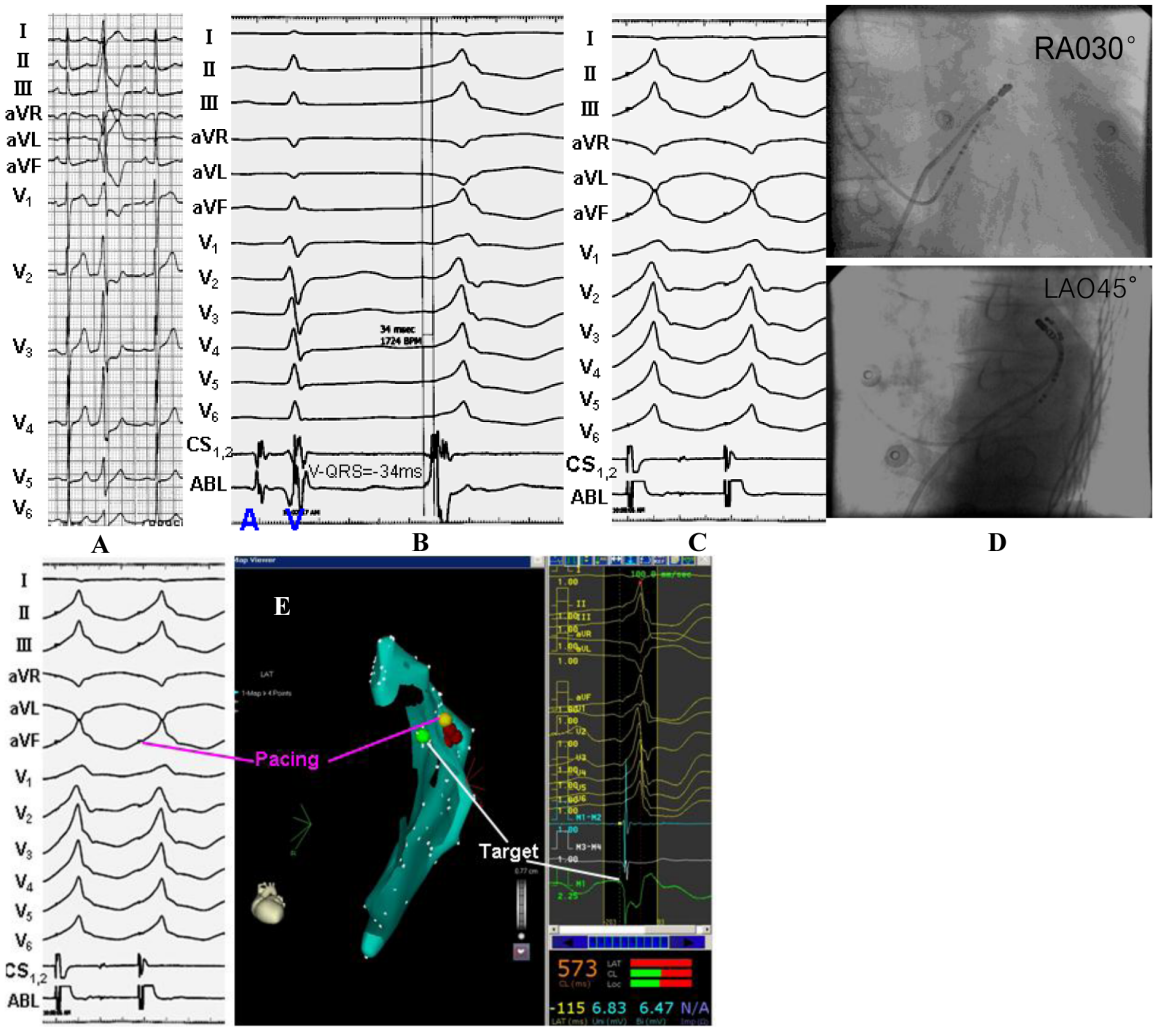
Example of a successful ablation of idiopathic premature ventricular contraction originating from the anterior portion of the epicardial mitral annulus. (A) The surface ECG characteristic. (B) The local ventricular activation time recorded at the successful ablation site that preceded the onset of the QRS complex was 34 ms. (C) Pace map at the ablation site. (D) The ﬂuoroscopic position of the ablation catheter site. (E) Green dot indicates site of RF application under the guide of Carto XP system.

All PVCs/IVTs arising from the ENDO and EPI MA displayed a right bundle branch block morphology with predominantly positive R wave in lead V1 (Table 5 and Figure 2–5). The precordial R-wave transition occurred earlier than lead V1 in all patients with PVCs/IVTs arising from the ENDO and EPI MA. In leads V2-V6, an R wave was dominant in all patients. 95.2% (20/21) PVCs/IVTs had an Rs pattern in lead V6. The duration of the QRS complex of the PVCs/IVTs was 153.3±15.7 (range 118–173) ms. The PdW time, the IDT and the MDI were 54.1±15.5 (range 0–76) ms, 76.9±13.3 (range 52–102) ms, and 0.50±0.07 respectively.

**Table 5:** 12-lead ECG characteristics of PVCs/IVTs originating from the endocardial and epicardial mitral annulus.

Pt	MA origin	QRS axis	QRS complex morphology												Trans- ition	PdW (ms)	IDT (ms)	QRS (ms)
			V1	V2	V3	V4	V5	V6	I	aVR	aVL	II	III	aVF				
1	AL-EP	right	R	Rs	Rs	Rs	Rs	Rs	rS	QS	QS	R	R	R	<V1	76	96	173
2	AL-EP	right	R	Rs	Rs	Rs	Rs	Rs	rS	QS	QS	R	R	R	<V1	61	78	150
3	AL-EP	right	R	Rs	Rs	Rs	Rs	Rs	rS	QS	QS	R	R	R	<V1	69	84	166
4	AL-EP	right	R	Rs	Rs	Rs	Rs	Rs	rS	QS	QS	R	R	R	<V1	63	95	169
5	AL-EP	right	R	Rs	Rs	Rs	Rs	Rs	rS	QS	QS	R	R	R	<V1	67	85	161
6	AL-EP	right	Rs	Rs	Rs	Rs	Rs	Rs	rS	QS	QS	R	R	R	<V1	68	86	164
7	AL-EP	right	Rs	Rs	Rs	Rs	Rs	Rs	rS	QS	QS	R	R	R	<V1	57	77	152
8	AL-EP	right	R	R	R	Rs	Rs	Rs	rS	QS	QS	R	R	R	<V1	51	82	154
9	PL-EP	left	R	Rs	Rs	Rs	Rs	Rs	qRs	r	R	rS	rS	rS	<V1	66	87	168
10	PS-EP	left	R	R	R	Rs	Rs	Rs	R	R	R	QS	QS	QS	<V1	54	102	146
11	PS-EN	left	qR	R	R	Rs	Rs	Rs	R	QS	R	rs	rS	rS	<V1	42	91	164
12	PS-EN	left	qR	R	R	Rs	Rs	Rs	R	qr	R	rS	rS	rS	<V1	46	70	163
13	AL-EN	right	Rs	Rs	Rs	Rs	Rs	Rs	rS	QS	QS	R	R	R	<V1	50	63	125
14	AL-EN	right	R	Rs	Rs	Rs	Rs	Rs	rs	QS	QS	R	R	R	<V1	48	60	153
15	PL-EN	normal	rsR′	R	R	R	R	R	R	QS	qr	Rs	rs	Rs	<V1	62	73	163
16	AL-EN	right	R	Rs	Rs	Rs	Rs	Rs	rS	QS	QS	R	R	R	<V1	51	67	118
17	AL-EN	right	R	Rs	Rs	Rs	Rs	Rs	qr	QS	QS	R	R	R	<V1	63	71	173
18	AL-EN	right	R	Rs	Rs	Rs	Rs	Rs	rS	QS	QS	R	R	R	<V1	45	63	129
19	MS-EN	left	qR	R	R	R	Rs	Rs	R	qr	R	rS	rS	rS	<V1	52	69	150
20	AL-EN	right	R	Rs	Rs	Rs	Rs	Rs	rS	QS	QS	R	R	R	<V1	46	52	142
21	AL-EN	right	R	Rs	Rs	Rs	Rs	Rs	rS	QS	QS	R	R	R	<V1	0	65	138

Capital letters (Q,R,S) refer to relatively high-amplitude waves (>5 mm). Conversely, lowercase letters (q,r,s) refer to relatively low-amplitude waves(≤5 mm). MA: mitral annulus; Transition: the site of R-wave transition in the precordial leads; PdW: pseudo delta wave time; IDT: intrinsicoid deflection time; QRS: duration of the QRS complex; AL-EP MA: anterolateral portion of the epicardial MA; PL-EP MA: posterolateral portion of the epicardial MA; PS-EP MA: posteroseptal portion of the epicardial MA; PS-EN MA: posteroseptal portion of the endocardial MA; AL-EN MA: anterolateral portion of the endocardial MA; PL-EN MA: posterolateral portion of the endocardial MA; MS-EN MA: midseptal portion of the endocardial MA.

#### ECG characteristics of PVCs/IVTs originating from the different portions of the MA

A monophasic R pattern was recorded in all three inferior leads (II, III, aVF) in all patients with PVCs/IVTs arising from the anterolateral portion of the ENDO and EPI MA (Tables 5 and 6), whereas a negative component (any s or S) was recorded in three inferior leads in all patients (5/5) with PVCs/VTs arising from the posterolateral and posteroseptal portion of the ENDO and EPI MA. A predominantly negative S pattern was recorded in lead I in all patients with PVCs/IVTs arising from the anterolateral portion of the ENDO and EPI MA, whereas a predominantly positive R wave in lead I was funded in 100% of patients (5/5) with PVCs/IVTs from the posterolateral and posteroseptal portion of the MA (Tables 5 and 6). When the origin of the PVCs/IVTs shifted from anterolateral to posterolateral to posteroseptal portion of the MA (Figure 2): R wave amplitude decreased and S wave amplitude increased in all inferior leads; R wave amplitude increased and S wave amplitude decreased in lead I. All 15 patients with PVCs/VTs arising from the anterolateral portion of the ENDO and EPI MA presented “notching” of the downstroke of the R wave in the inferior leads, but no “notching” of the downstroke of the R wave were found in PVCs/VTs arising from the posterolateral and posteroseptal portion of the ENDO and EPI MA (Table 6).

**Table 6:** Comparision of the QRS complex morphology of PVCs/IVTs originating from the endocardial and epicardial mitral annulus.

Origin	n	I	Inferior Leads	aVR	aVL	V1	V6	Early “Notching” In the Inferior Leads	Late “Notching” In the Inferior Leads
EP-MA	10								
AL	8	rS	R	QS	QS	R or Rs	Rs	+	+
PL	1	qRs	rS	r	R	R	Rs	–	–
PS	1	R	QS	R	R	R	Rs	+	–
EN-MA	11								
AL	7	rS	R	QS	QS	R or Rs	Rs	–	+
PL	1	R	R(r) s	QS	qr	rsR′	R	+	–
MS	1	R	rS	qr	R	qR	Rs	–	–
PS	2	R	rS	qr or QS	R	qR	Rs	–	–

EP MA: epicardial mitral annulus; EN-MA: endocardia mitral annulus; AL: anterolateral portion; PL: posterolateral portion; MS: midseptal portion; PS: posteroseptal portion; Early “Notching” indicates a Notching in the upstroke of the R wave in the inferior leads; Late “Notching” indicates a Notching in the downstroke of the R wave in the inferior leads.

#### Comparison between PVCs/VTs arising from the ENDO and EPI MA

**Figure 6:**
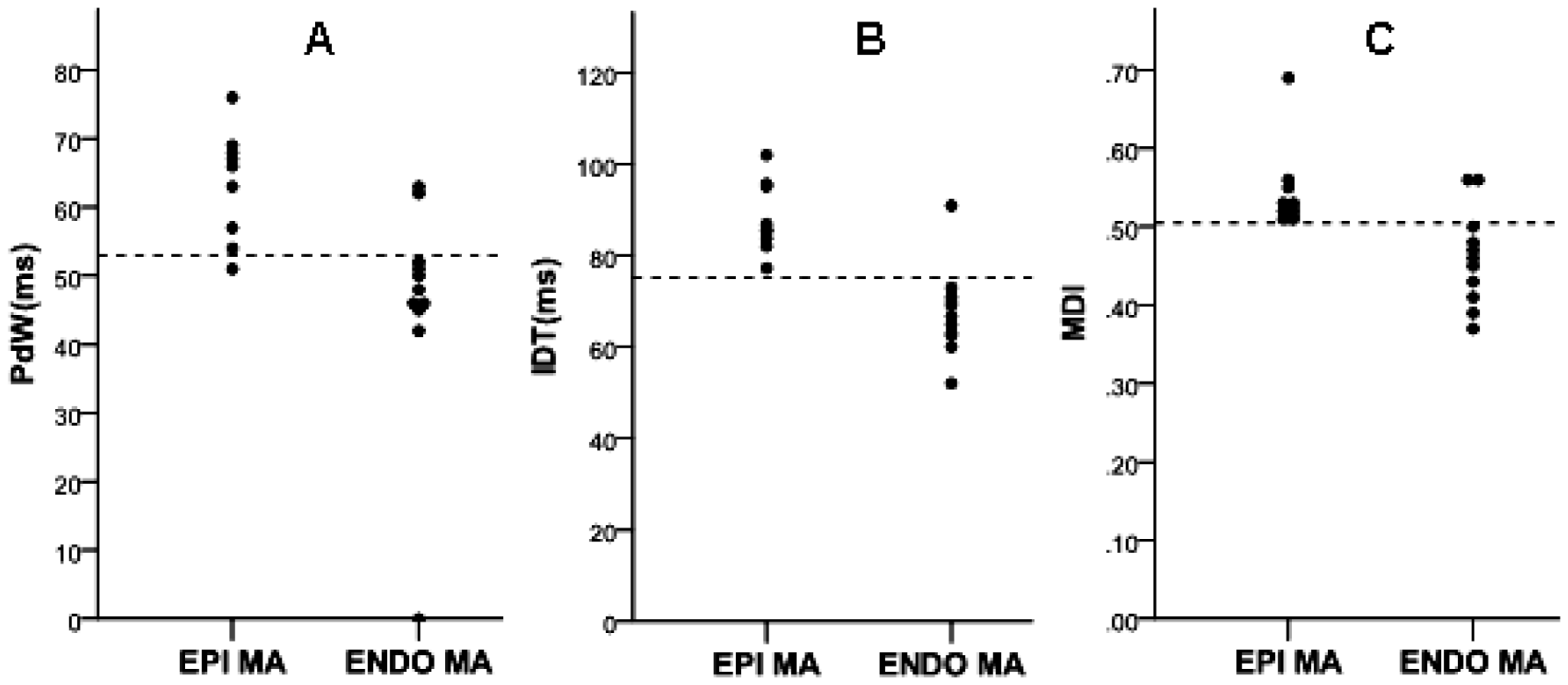
Distribution of PdW time (A) and precordial IDT (B) and MDI (C) values in patients with idiopathic PVC/VTs arising from the EPI and ENDO MA. The PdW time of >53 ms, the IDT time of >77 ms and MDI of >0.505 suggested the epicardial mitral annulus origin with high sensitivity and specificity. PdW: pseudo delta wave; IDT: intrinsicoid deflection time; MDI: maximum deflection index; EPI MA: epicardial mitral annulus; ENDO MA: endocardial mitral annulus.

The characteristics of the QRS morphology of the PVCs/IVTs arising from the EPI MA were almost identical with those of the PVCs/IVTs arising from the ENDO MA (Table 5). The QRS duration in PVCs/IVTs arising from the EPI MA were significantly longer compared with the arrhythmias arising from the ENDO MA (160.29±9.17 ms VS 147.09±18.08 ms, P<0.01). The PdW time and precordial IDT and MDI were significantly longer in patients with PVCs/IVTs arising from the EPI MA compared with those with PVCs/IVTs arising from the ENDO MA (63.20±7.60 ms VS 45.91±16.61 ms, P<0.01; 87.18±8.03 ms VS 67.55±9.73, P<0.01; 0.54±0.05 VS 0.46±0.06 P<0.05, respectively). The PdW time of >53 ms has a sensitivity of 90%, a specificity of 81.82%, a negative predictive value of 90%, and a positive predictive value 81.82%, the IDT time of >77 ms has a sensitivity of 100%, a specificity of 98.57%, a negative predictive value of 100%, and a positive predictive value 90.91%, and the MDI of >0.505 has a sensitivity of 100%, a specificity of 92.86%, a negative predictive value of 100%, and a positive predictive value 66.67% to predict the EPI MA origin of PVCs/IVTs (Figure 6).

#### Comparison between PVCs/IVTs arising from the anterolateral portion of the ENDO and EPI MA

**Figure 7:**
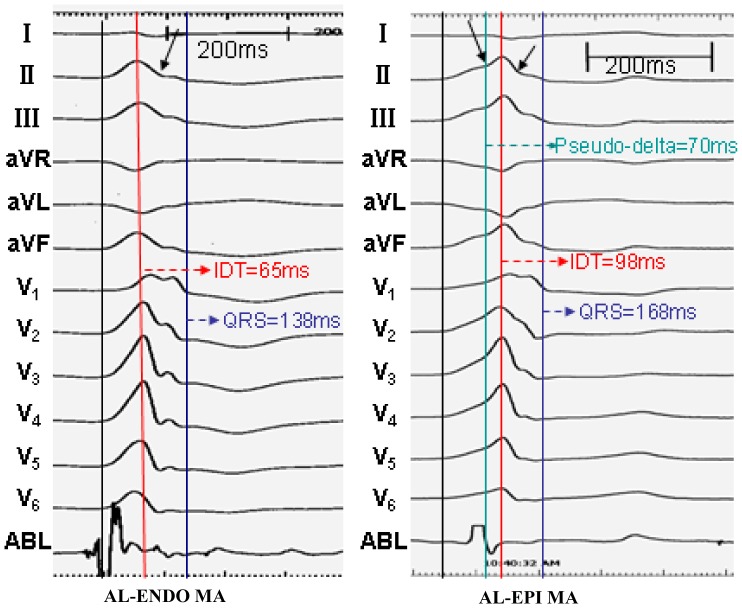
Example of intervals measured in premature ventricular contractions originating from AL-ENDO MA and AL-EPI MA. The PVCs/IVTs arising from the AL-EPI MA showed the “notching” (arrow) in both the upstroke and downstroke of the R wave in the inferior leads, but only the “notching” (arrow) of the downstroke of the R wave was found in PVCs/VTs arising from the AL-ENDO MA. The pseudo delta wave time and precordial IDT and MDI were significantly longer in patients with PVCs/VTs arising from the AL-EPI MA compared with that arising from the AL-ENDO MA.

**Figure 8:**
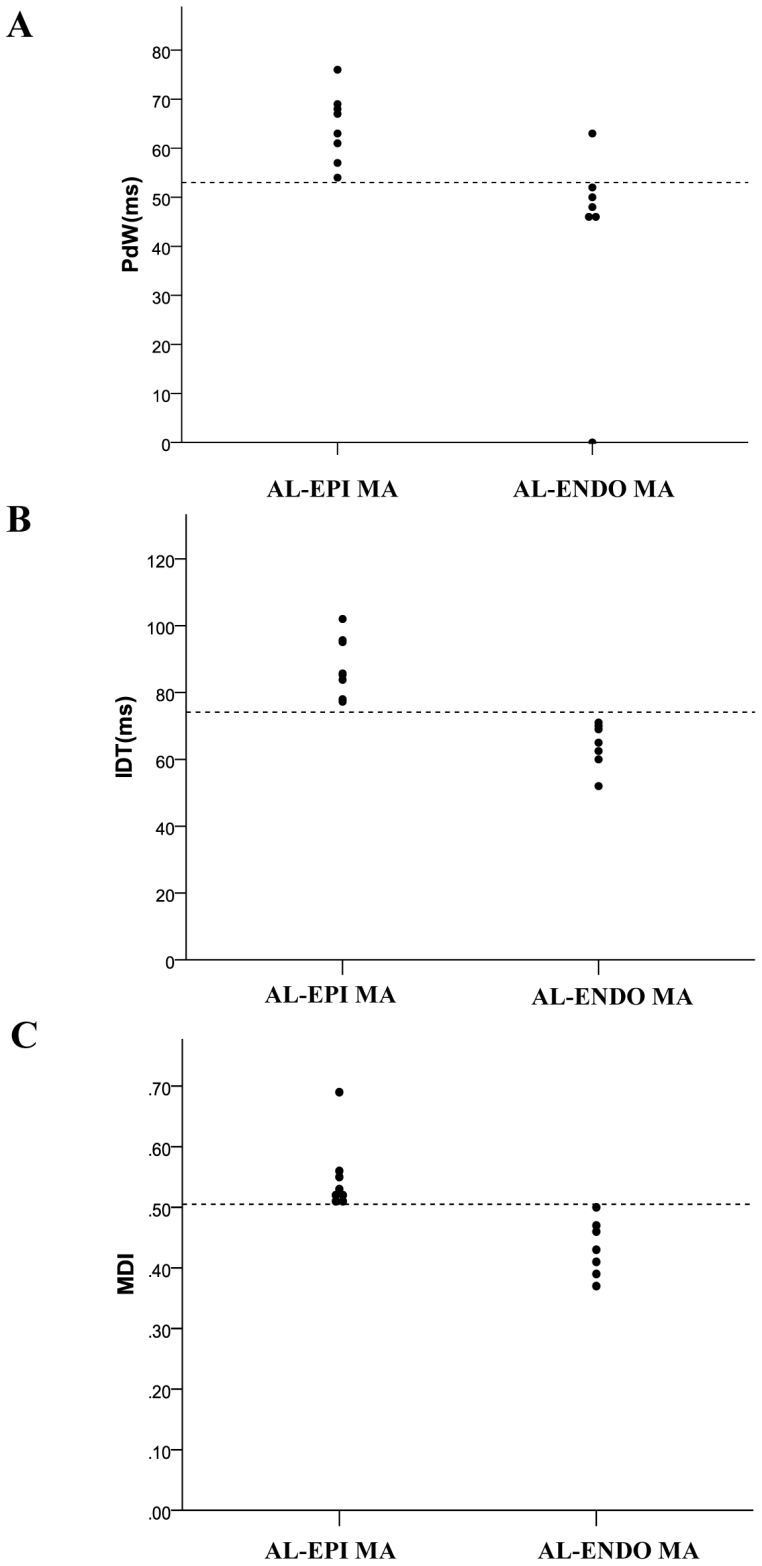
Distribution of PdW (A), precordial IDT (B) and MDI (C) values in PVCs/VTs arising from AL-ENDO MA and AL-EPI MA. The PdW time of >53 ms, the IDT time of >74 ms and MDI of >0.505 suggested the epicardial mitral annulus (EPI MA) origin with high sensitivity and specificity. PdW: pseudo delta wave; IDT: intrinsicoid deflection time; MDI: maximum deflection index; AL-EPI MA: anterolateral portion of EPI MA; AL-ENDO MA: anterolateral portion of endocardial MA.

Among the 21 patients with PVCs/IVTs arising from the vicinity of MA, PVCs/IVTs arise from the anterolateral portion of the MA in 15 patients, including 8 from the EPI MA and 7 from the ENDO MA. The anterolateral portion of the MA was the preferential site of origin (71.4%). There were no significant differences for the QRS complex morphology during PVCs/IVTs in 12 leads when PVCs/IVTs arising from the anterolateral portion of the EPI MA (AL-EPI MA) was compared with that arising from the anterolateral portion of the ENDO MA (AL-ENDO- MA). All PVCs/IVTs arising from the AL-ENDO MA and AL-EPI MA demonstrated a monophasic R pattern in all inferior leads, a QS pattern in leads aVR and aVL, a monophasic R or Rs pattern in lead V1, and an Rs pattern in leads V2-V6. All PVCs/IVTs arising from the AL-EPI MA showed the “notching” in both the upstroke and downstroke of the R wave in the inferior leads, but only the “notching” of the downstroke of the R wave was found in PVCs/VTs arising from the AL-ENDO MA (Table 6 and Figure 7). No significant differences in QRS duration were found when PVCs/VTs arising from the AL-EPI MA were compared with that arising from the AL-ENDO MA. The PdW time and precordial IDT and MDI were significantly longer in patients with PVCs/VTs arising from the AL-EPI MA compared with that arising from the AL-ENDO MA (64.38±7.09 VS 43.57±20.08, P<0.05; 87.85±8.87 VS 64.21±6.74, P<0.01; 0.55±0.06 VS 0.43±0.05 P<0.01, respectively). The “notching” in both the upstroke and downstroke of the R wave has a sensitivity of 100%, a specificity of 100%, a negative predictive value of 100%, and a positive predictive value 100%, the PdW time of >53 ms has a sensitivity of 100%, a specificity of 85.71%, a negative predictive value of 100%, and a positive predictive value 87.50%, the IDT time of >77 ms has a sensitivity of 100%, a specificity of 100%, a negative predictive value of 100%, and a positive predictive value 100%, and the MDI of >0.505 has a sensitivity of 100%, a specificity of 100%, a negative predictive value of 100%, and a positive predictive value100% to predict the AL-EPI MA origin of PVCs/IVTs (Figure 8).

### Pace mapping study

**Figure 9:**
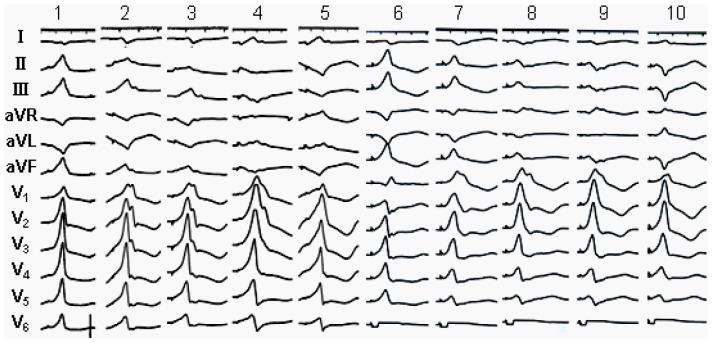
Pace mapping from the different portions of the mitral annulus (MA). 1, the anterior portion of the epicardial MA; 2, the anterolateral portion of the epicardial MA; 3, the lateral portion of the epicardial MA; 4: the posterolateral portion of the epicardial MA; 5: the posteroseptal portion of the epicardial MA; 6, the anterior portion of the endocardial MA; 7, the anterolateral portion of the endocardial MA; 8, the lateral portion of the endocardial MA; 9: the posterolateral portion of the endocardial MA; 10: the posteroseptal portion of the endocardial MA.

The pace mapping was performed from the 5 portions (anterior, anterolateral, lateral, posterolateral, and posteroseptal portions) along the ENDO and EPI MA. The characteristics of the QRS morphology during pacing from the ENDO and EPI MA in 12 subjects were almost identical with those of the PVCs/IVTs arising from the ENDO and EPI MA (Figure 9). There existed some distinctive ECG characteristics during pacing from the ENDO and EPI MA in 12 subjects (Figure 9): 1) During pacing from anterior to anterolateral to lateral to posterolateral to posteroseptal portions of MA, R wave amplitude was more lessening and S wave amplitude was more increasing in leads II, III, aVF. 2) During pacing from anterior to anterolateral to lateral to posterolateral to posteroseptal portions of MA, the QRS morphology progressively changed from QS pattern to qR pattern to R pattern in leads aVR and aVL, and from rS pattern to Rs pattern to R pattern in lead I. 3) A monophasic R or Rs pattern in lead V1 and an Rs or RS pattern in leads V2–V6 were always observed and the precordial R-wave transition always occurred earlier than lead V1 during pacing at the 5 portions of the ENDO and EPI MA. 4) The QRS duration were significantly longer during pacing from the EPI MA than during pacing from the ENDO MA (P<0.05), and the “notching” in both the upstroke and downstroke of the R wave in the inferior leads was always observed during pacing from the AL-EPI MA, but only the “notching” of the downstroke of the R wave was found during pacing from the AL-ENDO MA.

### Follow-Up

Patients have been followed-up for 18.2±12.6 (range 3–51) months. One patient with EPI MA VAs had recurrent VAs after an initially effective procedure. The transthoracic echocardiography demonstrated a decreased LVEDd in the 3 patients whose LVEDd was increased prior to ablation (Patients No. 4, 15, 20 in Table 1) and an increased LVEF (Patients No. 4, 9, 20 in Table 1) 3 months after effective RFCA. No patient died during follow-up.

## Discussion

### Main Findings

Several previous studies of idiopathic MA VAs have been limited because those studies did not include EPI MA VAs. This study was designed to overcome the limitation and revealed several novel findings as compared with prior reports. First, 3.52% of PVCs/IVTs had an origin at the ENDO and EPI MA. Second, the EPI MA VAs were as common as the ENDO MA VAs, and the MA VAs more commonly originated from the anterolateral portions than from the posterolateral and posteroseptal portions. Third, although the characteristics of the QRS morphology of the EPI MA VAs were almost identical with the ENDO MA VAs, The PdW time, IDT time and MDI were useful for differentiating EPI MA VAs from ENDO MA VAs. Finally, ablation via a transvenous approach is feasible and often effective for EPI MA VAs.

### Distribution of Idiopathic VAs in MA

Tada et al. have shown that most of idiopathic PVCs/VTs originating from the MA have their origin in the AL site of the MA [6]. In our series 71.4 percent of patients (15 of 21 patients) with MA VAs had the arrhythmia originating from the AL site of the MA. The AL portion of the MA was the preferential site of origin, which was similar to that of previously reported study [6]. Although several studies [5], [6] have determined the distribution of the ENDO site of origin, there are few studies evaluating the distribution of EPI sites of idiopathic MA VAs. In the present study, 48 percent of patients (10 of 21 patients) with MA VAs originated from the EPI MA. Idiopathic PVCs/VTs arising from the EPI MA may be more common than previously recognized. The present study indicates a wider distribution of sites of origin in the vicinity of MA.

### ECG Features

Prior studies [5], [6] have reported the ECG characteristics of VAs originating from the ENDO MA in patients without structural heart disease as the following: S or s waves in lead V6, “notching” of the late phase of the QRS complex in the inferior leads, and an early precordial tranditional zone (<V2). Site-specific criteria also have been described by these studies [5], [6]. Our results are similar regarding the characteristics of the QRS morphology of PVCs/IVTs arising from the ENDO MA. Although these ECG characteristics of MA VAs were helpful for differentiation from the other sites of PVCs/IVTs origin, the ECG characteristics predicting the EPI MA origin have not been studied. In the present study, we found that the ECG morphology of EPI MA VAs was similar with those of ENDO MA VAs. It is difficult to differentiate the ENDO and EPI MA origins because they both exhibit similar QRS morphologies. In this study, the prolonged PdW time and IDT time and precordial MDI, reflecting delayed initial activation of the left ventricle, reliably differentiated EPI MA VAs from ENDO MA VAs with high sensitivity and specificity.

### Radiofrequency Ablation

Previous studies have shown that PVCs/IVTs originating from the ENDO MA can be successfully ablated [5], [6]. In the present study, we demonstrated that RFCA was effective for eliminating PVCs/IVTs arising from the ENDO and EPI MA. Eighteen of 21 patients with PVCs/IVTs arising from the RV septum were successfully ablated (85.7% acute success). In 1 of the remain 3 patients, no attempt at ablation was undertaken because the origin of the PVCs/IVTs was found to be parahisian, and RF applications within coronary venous system were limited because there was too high impedance in the other 2 patients. One patient with EPI MA VAs had recurrent ventricular arrhythmia after successful RFCA during a mean follow-up period of 18.2 months. No significant complications were observed in our patient group confirming the safety of the procedure. Therefore, when the MA PVCs/IVTs can not be abolished by RF application from the endocardium, access via the CS for mapping and ablation of PVCs/IVTs sometimes is a good route, especially when the ECG features suggest an epicardial MA circuit. Although clinical application of RF energy in the CS has proven safe, much care must be taken to avoid the complications [8]–[11]. Coronary angiography performed prior to RF application may be useful for assessing the proximity of the coronary arteries to the proposed ablation site. In addition, it is important to minimize the amount of RF energy delivered from within the CS. If the pericardial circuit is not reachable via CS due to anatomical limitations, an alternative approach is via the pericardial space [13]. In addition, in this study, acute success was obtained in 14 of 15 patients using three-dimensional mapping system (93.3% acute procedural success; Table 4) and in 4 of 6 patients using conventional fluoroscopy guided mapping techniques (66.7% acute procedural success; Table 4). Freedom of PVCs/IVTs at follow up was obtained in 100% of patients using three-dimensional mapping and in 50% of patients using conventional mapping. Both acute and follow up results were comparable in the patients using three-dimensional mapping and conventional mapping (p = 0.184 and 0.222 respectively; Table 4). Therefore, three-dimensional mapping might raise acute and long term success in patients with PVCs/IVTs arising from the ENDO and EPI MA.

## Conclusions

We identified a group of patients with PVCs/IVTs arising from the ENDO and EPI MA. ECG characteristics of PVCs/VTs originating from the different portions of the MA are different, and can help regionalize the origin of these arrhythmias. RFCA at the ENDO MA or EPI MA via CS was relatively effective and safe for the PVCs/IVTs.
